# Tunneling nanotubes in microglia‐synapse crosstalk and inflammation

**DOI:** 10.1002/alz70855_102358

**Published:** 2025-12-23

**Authors:** Colleen R Zaccard, Euan Parnell, Marc Dos Santos, Skyler Zur, Isabel Gippo, Blair L Eckman, Changiz Geula, Peter Penzes

**Affiliations:** ^1^ Northwestern University Feinberg School of Medicine, Chicago, IL, USA; ^2^ Mesulam Center for Cognitive Neurology & Alzheimer's Disease, Chicago, IL, USA; ^3^ Northwestern ADC Neuropathology Core, Northwestern University Feinberg School of Medicine, Chicago, IL, USA

## Abstract

**Background:**

Microglia, the brain's innate immune cells, regulate synaptic function by remodeling postsynaptic spines or pruning complement‐tagged presynaptic terminals via complement receptors (CRs). In Alzheimer's disease (AD), microglia dysfunction drives chronic inflammation and synapse loss through aberrant pruning and unknown mechanisms. Tunneling nanotubes (TNTs) are long, ultrafine membranous connections that facilitate rapid cell‐cell communication. TNTs have been implicated in neuro‐immune crosstalk but remain understudied due to their nanoscale and lack of specific markers. Plasma membrane‐organizing tetraspanins CD9 and CD81 are enriched in the TNT proteome of cell lines and regulate TNT stability and vesicle trafficking, suggesting their utility for TNT‐specific labeling.

**Method:**

Human primary microglia, isolated postmortem from cognitively normal elderly, were transduced with a tdTomato (tdT) AAV to visualize TNTs. Microglia were polarized for 24 hours with vehicle (control), oligomeric amyloid‐beta (oAβ) + IFN‐γ (pro‐inflammatory), or IL‐4 + IL‐13 (anti‐inflammatory) cocktails. Immuno‐labeled CR3/CR4 localization to TNTs versus cell bodies was assessed using enhanced resolution (ER) confocal microscopy. Similarly, microglia were treated with 5FAM‐oAβ + vehicle or IFN‐γ to assess oAβ localization to TNTs during inflammation. Microglial TNTs were analyzed in live microglia‐excitatory neuron (iEN) co‐cultures under control, pro‐inflammatory, and anti‐inflammatory conditions. Finally, CD9 and CD81 were evaluated as candidate microglial TNT markers using ER confocal and super‐resolution microscopy.

**Result:**

Pro‐inflammatory conditions significantly increased TNT number per microglia and enhanced CR3/CR4 localization on TNTs compared to cell bodies, suggesting complement‐dependent functions. Pro‐inflammatory cues elevated oAβ localization to TNTs, suggesting roles in pathological protein trafficking. In live microglia‐iEN co‐cultures, microglial TNT frequency, length, and dynamic interactions with axons, dendrites, and presumptive spines were enhanced in response to inflammation. CD81 particularly localized to microglial TNTs under varied conditions, suggesting its utility as a TNT‐specific marker *in vivo*.

**Conclusion:**

Microglia respond to AD‐relevant pro‐inflammatory cues with increased TNT expression, CR and oAβ localization to TNTs, and enhanced TNT‐mediated interactions with synaptic elements. Thus, TNT composition and functions are environmental context dependent. Inflammation‐inducible TNTs may participate in complement‐dependent synaptic remodeling and/or pathological protein trafficking. CD81 represents a promising marker for in vivo investigations of the microglial TNT‐synapse interface during chronic inflammation, aging, and AD.

